# Surface-enhanced Raman scattering as a potential strategy for wearable flexible sensing and point-of-care testing non-invasive medical diagnosis

**DOI:** 10.3389/fchem.2022.1060322

**Published:** 2022-11-03

**Authors:** Guoran Liu, Zhimei Mu, Jing Guo, Ke Shan, Xiaoyi Shang, Jing Yu, Xiu Liang

**Affiliations:** ^1^ Advanced Materials Institute, Qilu University of Technology (Shandong Academy of Sciences), Jinan, China; ^2^ Shandong Artificial Intelligence Institute, Qilu University of Technology (Shandong Academy of Sciences), Jinan, China; ^3^ School of Physics and Electronics, Shandong Provincial Engineering and Technical Center of Light Manipulation, Shandong Normal University, Jinan, China

**Keywords:** SERS, flexible wearable sensor, non-invasive medical diagnosis, POCT, PIC

## Abstract

As a powerful and effective analytical tool, surface-enhanced Raman scattering (SERS) has attracted considerable research interest in the fields of wearable flexible sensing and non-invasive point-of-care testing (POCT) medical diagnosis. In this mini-review, we briefly summarize the design strategy, the development progress of wearable SERS sensors and its applications in this field. We present SERS substrate analysis of material design requirements for wearable sensors and highlight the benefits of novel plasmonic particle-in-cavity (PIC)-based nanostructures for flexible SERS sensors, as well as the unique interfacial adhesion effect and excellent mechanical properties of natural silk fibroin (SF) derived from natural cocoons, indicating promising futures for applications in the field of flexible electronic, optical, and electrical sensors. Additionally, SERS wearable sensors have shown great potential in the fields of different disease markers as well as in the diagnosis testing for COVID-19. Finally, the current challenges in this field are pointed out, as well as the promising prospects of combining SERS wearable sensors with other portable health monitoring systems for POCT medical diagnosis in the future.

## Introduction

Wearable sensor technology is an essential link in the future of customized medicine, which is crucial for early disease diagnosis, cancer biomarker identification, biological monitoring, and treatment (Cheng, et al., 2021a; Liu, et al., 2021). Such sensors must overcome the mismatch between traditional rigid silicon-based sensors and soft, flexible organisms to fit perfectly at biological interfaces, such as the epidermis, eyes, and teeth, to assess human health conditions (Gao, et al., 2016; Peng, et al., 2020; Yang, et al., 2020). Current wearable sensors cannot accurately detect low concentrations of analytes, lack multimodal sensing, and typically can only detect one analyte at a time. Therefore, developing flexible wearable sensors with ultra-sensitive and multifunctional detection abilities is still a challenge.

Surface-enhanced Raman scattering (SERS) has attracted considerable interest from researchers in the fields of wearable flexible sensing and point-of-care testing (POCT) for non-invasive medical diagnosis. The SERS system consists of a probe and substrate. The probe, as the enhanced molecule, is also the analytical object of Raman spectroscopy. The substrate is the material used for enhancement, which can be either a roughened metal electrode or a metallic nanomaterial. SERS technology enables Raman signal enhancement of probe molecules by adsorbing the probe to the substrate surface (Fleischmann, et al., 1974; Albrecht and Creighton 1977; Jeanmaire, et al., 1977; Ding, et al., 2020). The fundamental prerequisite for improving SERS technology from qualitative to quantitative analysis depends on the homogeneity, sensitivity, stability, and university of SERS substrates (Liang, et al., 2015; Li, et al., 2018; Liang, et al., 2021; Lyu, et al., 2023). The three-dimensional (3D) periodic SERS array structure is able to balance homogeneity and enhancement, providing a large number of nanogaps in SERS substrate for inducing the increase of local photonic state density as well as creating more spatial hot spots for SERS activity enhancement (Jung, et al., 2014; Liu, et al., 2014; Zhang, et al., 2014; Koh, et al., 2021). Most importantly, the periodic array system provides high signal reproducibility and reliability, ensuring the uniformity of SERS substrates on the whole surface, which is also an important means to solve the application limitations of SERS substrates (Liang, et al., 2017; Liang, et al., 2022; Zhang, et al., 2022). A well-established SERS substrate with biocompatibility, high sensitivity, and stable monitoring capability is essential for rapid, safe, and practical *in situ* POCT wearable sensing analysis (Yang, et al., 2018).

Since the concept of SERS firstly proposed in 1977 (Albrecht and Creighton 1977; Jeanmaire, et al., 1977), SERS has experienced significant growth in both basic and applied research. Great progress has been made in POCT non-invasive medical diagnostics based on different materials or structures of SERS sensors. For example, paper-based SERS plasmonic sensors can sensitively detect and quantify uric acid in sweat at concentrations as low as 1 µM for the diagnosis of cardiovascular disease, kidney disease, and type Ⅱ diabetes (Mogera, et al., 2022); Sensors using binary nanosphere arrays (SiO_2_@Au@AuNPs, SAAs) as SERS substrates can detect bilirubin in tears to diagnose jaundice (Zhao, et al., 2022). The wearable SERS sensor developed by using the omnidirectional plasmonic nanogap arrays (OPNA) can quantitatively detect dopamine (DA) in sweat for monitoring neurological disorders or emotional activity (Zhu, et al., 2022).

In view of the above research background and significance, this mini-review mainly outlines the design principles of the new flexible SERS sensor substrate, the latest research progress, and its application research in non-invasive POCT medical diagnosis, and looks forward to the great application prospects of SERS wearable sensors in the fields of different disease markers such as cancer markers, coronavirus disease 2019 (COVID-19) detection, etc. Its association with other health monitoring wearable medical portable devices will further promote the rapid development and application of personalized medicine, which has important research significance and application value for the diverse sensing needs of remote personal health monitoring system.

## Wearable surface-enhanced Raman scattering sensors：Design and development

**FIGURE 1:**
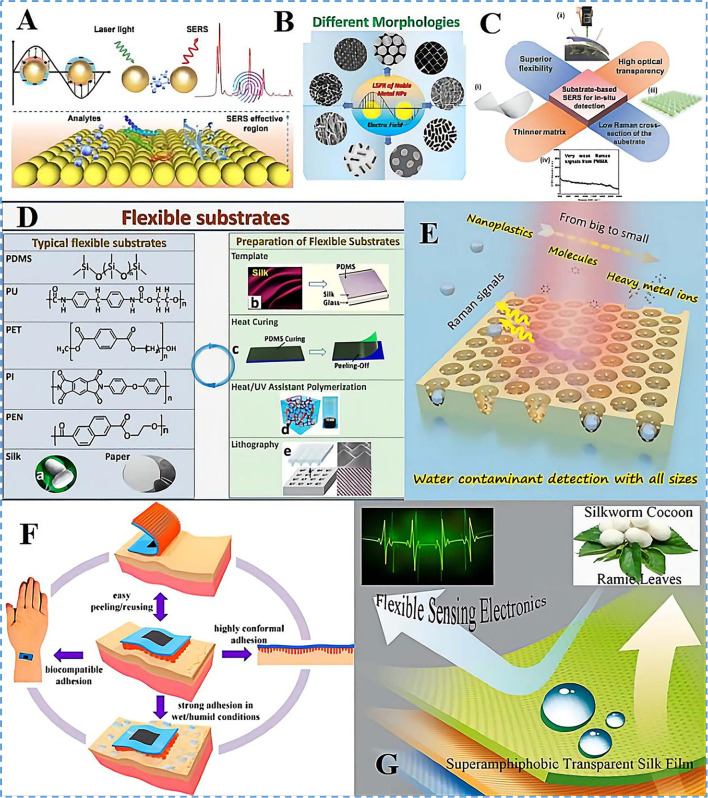
Wearable SERS sensor design and development. **(A)** Schematic diagram of the principle of SERS detection analyte. **(B)** localized surface plasmon resonance (LSPR) effects based on noble metal nanoparticles with different morphologies. Copyright^©^ Tsinghua University Press and Springer-Verlag GmbH Germany, part of Springer Nature 2021. **(C)** The crucial features of substrate-based SERS for *in situ* detection. (i-ii) Flexible materials and excitation laser lasers. (iii) SERS substrates. Copyright^©^ 2017, American Chemical Society. (iv) Raman signals from polymethyl methacrylate. Copyright^©^ 2014, American Chemical Society. **(D)** Typical types of flexible substrates and preparation methods in flexible SERS substrates: **(A)**. Silkworm cocoon optical photo; **(B)**. Preparation of flexible substrates by stencil method; **(C)**. Preparation of flexible substrates by thermosetting spinning; **(D)**. Preparation of flexible substrates by heat/UV-assisted polymerization; **(E)**. Preparation of flexible substrates by a photolithography stencil method. Reprinted (adapted) with permission from. Copyright^©^ 2021 American Chemical Society. **(E)** AAO/MoS2/Ag 3D PIC structure SERS substrate working principle and detection size. Reprinted (adapted) with permission from. Copyright^©^ 2022 American Chemical Society. **(F)** Characteristics of the MSFA material. Reprinted (adapted) with permission from. Copyright^©^ 2020 American Chemical Society. **(G)** An integrated silk membrane with special wettability shows excellent self-cleaning, transparency, and flexible sensing properties under harsh conditions. Reprinted (adapted) with permission from. Copyright^©^ 2020 American Chemical Society.

SERS is achieved by adsorbing probe molecules on the substrate surface (Zhang, et al., 2021). At present, the common enhancement techniques include chemical mechanisms derived from charge transfer resonance and electromagnetic mechanisms, as well as the combination of both mechanisms (Lee, et al., 2021). For the SERS mechanism, the localized surface plasmon resonance (LSPR) effect is the most significant one, as shown in Figure 1A. The LSPR effect takes place when the frequency of incident light coincides with the inherent oscillation frequency of the electrons in the plasmonic nanostructure. The LSPR effect is an electron density wave that propagates along the metal surface due to the interaction between free electrons on the surface of the noble metal nanostructures. As seen in Figure 1B, when the light interacts with the nanoparticle, the incident photons are scattered in different directions, which enhances the light field, electric field, and magnetic field, and increases scattering cross-sections of the sample to make Raman scattering possible (Li, et al., 2018; Lin, et al., 2018; Xie, et al., 2022). The LSPR performance of Raman enhanced nanoparticles directly affected the sensitivity, repeatability and stability of SERS, which determined the usefulness and reliability of SERS technology. The LSPR performance of nanoparticles is closely related to its size, morphology, distance and hot spot. For nanoparticles with different morphologies, their LSPR performance is different, resulting in the difference of SERS performance. For nanoparticles with the same morphology, the LSPR intensity is uniform at each site except for spherical, and the LSPR intensity at different sites of other morphologies such as nanorod, nanocube, nanoflower and nanosheet are different. When the incident light is excited from different angles, the same material will exhibit different properties, which will affect the repeatability of SERS signal.

Flexible SERS substrates can be attached to any surface due to their high flexibility, which circumvents the drawbacks of rigid SERS substrates in detection scenarios. As seen in Figure 1C, the following requirements must be met for *in situ* detection with flexible SERS substrates: 1) facile substrate deformation; 2) excellent light transmission; 3) ultra-thin substrate material and 4) low substrate interference with Raman signals (Xu, et al., 2019). Practical flexible SERS substrates just need to have high deformation and low interference Raman signal qualities to meet most of the detection requirements because most flexible substrates are unable to combine ultra-thin and light-transmitting properties.

Typical flexible SERS substrates include both noble metal and substrate components. The desired flexible substrate usually has one or more of the following characteristics: scalability, adhesion, biocompatibility, and stability (Koh, et al., 2021). The most widely used flexible substrate materials are polymer substrates. Polydimethylsiloxane (PDMS) has stable chemistry properties, good tensile and flexural elasticity (100–1100%), good thermal stability, transparency, and biocompatibility (Wang, et al., 2018). Fiber paper and silk fibroin (SF) (Sajal Kumar 2012) are also considered promising flexible substrates because of their high biocompatibility and biodegradability. Zhang’s group (Wang, et al., 2014) used the microstructure of the surface of silk fibroin to construct pattern-based flexible PDMS film substrates as shown in Figure 1D (Song, et al., 2021). The flexible substrates were prepared by using thermal curing and spin molding methods, while the hydrogels were polymerized under light or heat conditions with the aid of templates to produce flexible ultrathin substrates (Parolo and Merkoçi 2013). Considerable emphasis has been given to silk proteins, which are secure, non-toxic, immune-suppressive, and a naturally appealing biocompatible material, in adaptable electrical systems (Hu, et al., 2021). SF membranes show promise in flexible electronic, optical and electrical sensors due to their excellent mechanical properties. Li et al. prepared superhydrophobic, transparent and flexible smart filing protein membranes by spraying long silver nanowires (AgNWs) dispersed in polydimethylsiloxane (PDMS) followed by vacuum drying. The resulting SF/AgNWs membranes are super-repulsive to liquids with low surface tension and water. Due to the excellent mechanical properties of SF, it has tensile, bending, and high transparency stability. It can also be used for human motion detection under wet conditions as shown in Figure 1G (Li, et al., 2020). The transparent, flexible SERS substrate was developed by Liu et al. (2019) and generated by using a blotting method for electron substitution on a fibrin membrane. The nanocatheter, fabricated on a flexible filament-like fibrin network, overcomes the bending, cutting, remodeling, spatial processing and manipulation characteristics of traditional rigid materials. Multifunctional hybrid metal film material that can be used for bending optics, wearable medical diagnostic electronics, and recyclable catalysts. Besides, SF is made up of hydrophilic and hydrophobic amino acid sequences that give it good interfacial adhesion and excellent hygroscopic properties, producing a moisture-driven effect when it encounters water molecules (Huang and Chiu 2021; Manikandan, et al., 2021). A microstructured fibroin adhesive that can be used in wearable electronic devices was developed by Liu et al. (2020) Due to the unique interfacial adhesion effect, the prepared materials also have a high adhesive force in addition to their highly biocompatible and reusable properties, making them effective under wet conditions. And avoid the sharp pain feeling during the removal process, it can be used in skin-sensitive people and athletes as shown in Figure 1F.

Advances in nanofabrication technology allow particle-in-cavity (PIC) structures to generate extremely high field enhancements. To achieve high SERS sensitivity and uniformity, Wang et al. created a wearable SERS ultra-thin and flexible multi-functional sensor (SF-AAO-Au) based on a micro-nano array of cavity particle structure of silk fibroin, anodic aluminum oxide (AAO) and gold nanoparticles. The high SERS sensitivity and uniformity are due to the efficient enhancement ability of porous 3D PIC structure and highly ordered periodic array (Liu, et al., 2022; Wang, et al., 2022). As illustrated in Figure 1E, Li et al. (2022) designed a quasi-periodic PIC Raman substrate structure based on silver nanoparticles and anodic alumina templates to achieve sensitive detection at the single-molecule level and further improved the detection accuracy by introducing molybdenum disulfide as an internal standard. With the advantages of local hot spots, spatial hot spots and high adsorption properties of PIC structure intrinsic, the AAO/MoS_2_/Ag 3D structure achieved sensitive detection of multi-size pollutants including heavy metal ions, small molecule pollutants, and nano plastics in the water.

## Applications in point-of-care testing non-invasive medical diagnosis

The growing interest in the field of flexible SERS sensing research over the past few years has also driven the expansion of SERS technology from a laboratory research tool to a practical application. Emerging flexible SERS substrates are attracting unprecedented attention to meet the need for *in situ* and real-time monitoring for POCT diagnostics. Its applications mainly focus on preparing and applying transparent and flexible SERS active films (Xie, et al., 2022). Hong’s group (Xu, et al., 2019) highlighted three recent types of flexible SERS platforms including tunable SERS, swab sampling SERS, and *in situ* SERS. Sun’s group (Zhang, et al., 2021) focused on summarizing the development of flexible SERS substrates for non-destructive food detection. Liu et al. (2021) introduced three types of flexible SERS platforms and their practical applications including paper-based SERS substrates, polymer-based SERS substrates, and inorganic material-based SERS substrates.

**FIGURE 2:**
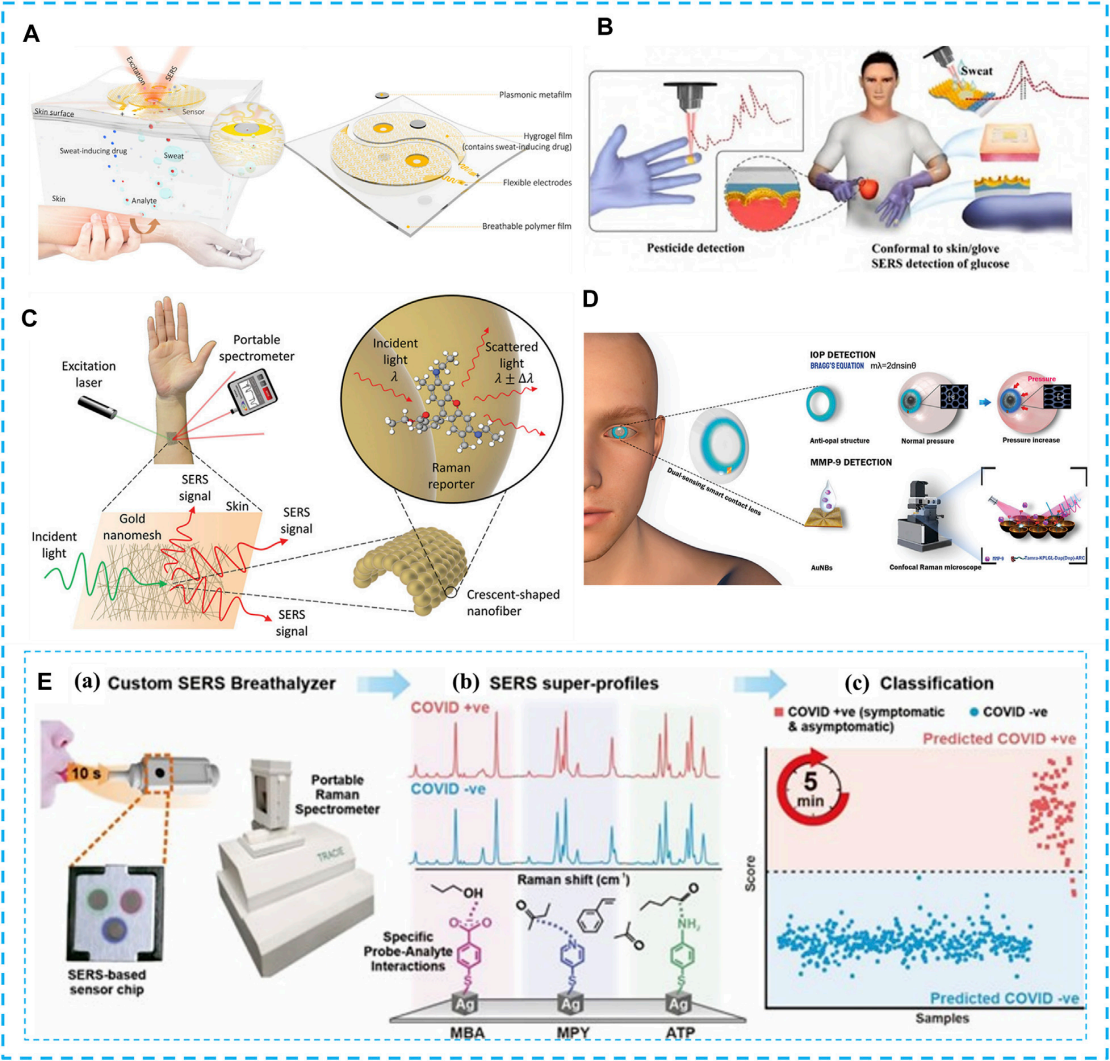
SERS non-invasive medical diagnosis. **(A)** Schematic diagram of the working principle and design of the plasma metamaterial integrated wearable SERS sensing device, consisting of two main components (sweat extraction component and SERS sensing component) with the appearance of a yin and yang symbol. Copyright^©^ 2021, American Association for the Advancement of Science. **(B)** Ultra-thin flexible SF-AAO-Au substrate pesticide detection lab gloves and sweat glucose dual function wearable SERS sensor. Copyright^©^ 2022, Elsevier. **(C)** Concept diagram of wearable skin surface SERS. Copyright^©^ 2022, Wiley. **(D)** Schematic diagram of a bifunctional contact lens sensor consisting of an anti-catenary structure for IOP monitoring and a peptide-functionalized AuNBs SERS substrate for MMP-9 detection. Copyright^©^ 2022, Wiley. **(E)** Overview of SERS-based strategy for identifying covid-positive individuals using respiratory volatile organic compounds (BVOCs): **(A)**. Breath testing using a handheld surface-enhanced Raman scattering-based breath detector for 10s blowing; **(B)**. Chemical interaction with BVOCs using MBA, MPY, and ATP as molecular receptors to bring gaseous analytes close to the plasma surface; **(C)**. Receive test results within 5 min. Copyright^©^ 2022 American Chemical Society.

Flexible SERS sensors have been used to diagnose and screen for various diseases such as cancer, diabetes and glaucoma by detecting disease-related biomarkers. Liu et al. (Cheng, et al., 2021b) introduced flexible plasma metasurface with SERS activity as a basic sensing component for wearable sensors (Figure 2A). Since sweat samples containing different drugs exhibit completely different SERS spectra, the sensor can fingerprint the targets extracted from sweat, monitor changes in trace amounts of drugs in the body through sweat, and obtain an individual drug metabolism profile. Wang et al. (2022) designed a bifunctional ultra-thin flexible PIC array structure-based SF-AAO-Au wearable SERS sensor with good skin adhesion, excellent mechanical flexibility, and SERS activity is not limited by any natural skin deformation (Figure 2B). The developed SF-AAO-Au SERS sensor shows excellent glucose recognition in the concentration range of 100–10^6^ nM with LOD down to 168 nM enabling non-destructive, painless and rapid qualitative detection of ultra-trace low concentrations of sweat glucose on real human skin surfaces. The wearable SERS sensor of Keisuke Goda et al. (Liu, et al., 2022) tested human sweat biomarkers: urea and ascorbic acid, which is capable of detecting and identifying low concentrations of different analytes on almost any arbitrary surface (Figure 2C), essential for a timely, accurate and comprehensive understanding of the wearer’s complex physiological and pathological condition. Wang et al. (Ye, et al., 2022) combined peptide-modified gold nanobowls (AuNBs) with SERS substrates to introduce a dual-functional smart contact lens sensor that enables continuous monitoring of intraocular pressure by observing structural color changes and quantitative SERS analysis of MMP-9 for the purpose of glaucoma diagnosis, effectively avoiding potential damage to the eye from electronic components (Figure 2D). Ling et al. (Leong, et al., 2022) designed a respiratory assay based on three separate sets of SERS probe molecules: 4-mercaptobenzoate (MBA), 4-mercaptopyridine (MPY), and 4-aminothiophenol (ATP), for rapid and non-invasive screening of COVID-19 individuals. Which can identify COVID-19-infected individuals within 5 min and achieve 96.2% sensitivity, and 99.9% specificity in 501 participants, advancing the next generation of non-invasive human respiratory diagnostic kits (Figure 2E).

## Conclusion and perspective

The mini-review mainly introduces the design and development of wearable SERS sensors and their application in POCT non-invasive diagnosis. In the design of wearable SERS sensors, the materials should guarantee stability, adhesion, scalability, sensitively, and biocompatibility based on excellent SERS performance. The work summarized several widely used flexible substrate materials and highlights the benefits of new plasma flexible SERS sensors based on PIC structures, as well as the unique interfacial adhesion effect and excellent mechanical properties of SF derived from natural cocoons, indicating promising futures for applications in the fields of flexible electronic, optical, and electrical sensors. Additionally, the application of flexible SERS substrates in POCT diagnostics is focused on the preparation and application of transparent, flexible SERS active films and their use in the diagnosis and screening of various diseases such as cancer, diabetes, and glaucoma. SERS wearable sensors have great potential for use in the field of different disease markers, as well as their application in the detection of COVID-19, which is widely dispersed throughout the world now. The rapid development and application in the area of customized medicine will be further promoted by the combination of SERS wearable sensors with other portable health monitoring systems. In the real-time monitoring of health-related target biomarkers sensitive analysis in the multiple sweat/tear/urine systems, challenge still exists in the aspects of improving anti-interference or selectivity of target biomarkers in complex human body fluids environment such as sweat, tears, urine or other liquids which including electrolytes (Na^+^, K^+^), glucose, lactic acid, protein, and other components. On the one hand, SERS technology can be combined with microfluidic system. Through the intelligent design of microfluidic system, different analytes can be oriented to different functional areas to achieve simultaneous separation and efficient analysis of multi-components. On the other hand, the SERS substrates can also be designed as effective separation filter membrane to separate body fluid molecules with different molecular sizes to achieve more selective and highly sensitive marker identification. Despite the current challenges in the design and application of multiple materials and the interference of impurities in complex samples, SERS is expected to make significant achievements in the field of POCT non-invasive diagnosis with the in-depth research and optimization of flexible materials by researchers.
